# Deubiquitinase OTUD7B stabilizes HNF4α to alleviate pressure overload-induced cardiac hypertrophy by regulating fatty acid oxidation and inhibiting ferroptosis

**DOI:** 10.1186/s40364-025-00766-2

**Published:** 2025-03-29

**Authors:** Rujie Zheng, Wenjuan Song, Che Wang, Xiaoyu Du, Chunlei Liu, Xiaotong Sun, Chengzhi Lu

**Affiliations:** 1 The First Central Clinical School, Tianjin Medical University, Tianjin, China; 2 School of Medicine, Nankai University, Tianjin, China; 3 Department of Cardiology, Tianjin First Central Hospital, 24 Fukang Road, Nankai District, Tianjin, 300192 People’s Republic of China

**Keywords:** Cardiac hypertrophy, Deubiquitinating enzyme, Ferroptosis, Fatty acid oxidation

## Abstract

**Background:**

Cardiac hypertrophy, a leading cause of heart failure, threatens global public health. Deubiquitinating enzymes (DUBs) are critical in cardiac pathophysiology by regulating protein stability, function, and degradation. Here, we investigated the role and regulating mechanism of ovarian tumor domain-containing 7B (OTUD7B) in cardiac hypertrophy by modulating fatty acid metabolism.

**Methods:**

Mice subjected to transverse aortic constriction (TAC) and cardiomyocytes treated with phenylephrine (PE) were used to explore the role of OTUD7B in myocardial hypertrophy. The potential molecular mechanisms underlying OTUD7B's regulation of cardiac hypertrophy were explored through transcriptome analysis and further validated in cardiomyocytes.

**Results:**

Reduced OTUD7B expression was observed in hypertrophic hearts following TAC surgery. Cardiac-specific OTUD7B deficiency exacerbated, while OTUD7B overexpression mitigated, pressure overload-induced hypertrophy and cardiac dysfunction both in vivo and in vitro. OTUD7B knockdown resulted in ferroptosis, as evidenced by decreased mitochondrial cristae, increased Fe^2+^ ion content, lipid peroxide accumulation, while OTUD7B overexpression inhibited ferroptosis. Mechanistically, transcriptomic analysis identified OTUD7B plays a role in the regulation of fatty acid metabolism and pathological cardiac hypertrophy. OTUD7B was found to directly bind to HNF4α, a transcription factor regulating fatty acid oxidation-related genes. Further, OTUD7B exerted deubiquitination activity to stabilize the HNF4α protein by removing K48-linked ubiquitin chains, thereby preventing its degradation via the proteasomal pathway and linking the HNF4α degradation and ferroptosis. Finally, ferroptosis inhibitors, ferrostatin-1, alleviated OTUD7B inhibition-induced ferroptosis, fatty acid metabolism suppression, and myocardial hypertrophy.

**Conclusions:**

We confirmed that OTUD7B is involved in the regulation of ferroptosis in pressure overload-induced cardiac hypertrophy and highlighted that OTUD7B alleviates cardiac hypertrophy by regulating ferroptosis and fatty acid oxidation through deubiquitination and stabilization of HNF4α.

**Supplementary Information:**

The online version contains supplementary material available at 10.1186/s40364-025-00766-2.

## Introduction

Cardiac hypertrophy represents a compensatory mechanism of the heart in response to increased workload caused by various pathological stimuli [1, 2]. While initially beneficial by enhancing cardiac output, sustained hypertrophy transitions into maladaptive remodeling, culminating in heart failure and irreversible cardiac dysfunction. The underlying mechanisms of cardiac hypertrophy are complex, involving changes in protein synthesis, mechanical stress, oxidative damage, and disruptions in cytoskeleton remodeling [3, 4]. Despite promising advances in the treatment of cardiac hypertrophy, such as the myosin inhibitor mavacamten significantly improving clinical symptoms [5], challenges remain. The use of mavacamten is limited by its potential to reduce left ventricular ejection fraction (LVEF), making it unsuitable for patients with LVEF < 50% [5]. Given the persistent challenge, deeply investigating the molecular and metabolic changes driving pathological cardiac remodeling and developing new approaches targeting key intracellular regulatory proteins and their post-translational modifications may offer innovative therapeutic strategies for mitigating the progression of hypertrophy-related cardiac diseases [4, 6].

Deubiquitinating enzymes (DUBs) have emerged as attractive therapeutic targets due to their involvement in modulating the stability of proteins that are crucial in hypertrophic cardiomyopathy and other cardiovascular disorders. Ubiquitination is a critical posttranslational modification involved in numerous cellular functions, such as signal transduction, protein turnover and cell fate determination [7–9]. This dynamic modification is regulated by DUBs, which reverse the attachment of ubiquitin to substrate proteins [10], ensuring proper protein stability and function.

The OTU (ovarian tumor-related proteases) family represents a distinct subfamily of deubiquitinating enzymes, characterized by the presence of a catalytic OTU domain, an alanine-rich region and a ubiquitin-interacting motif [11]. Recent studies have identified the involvement of OTU proteins in various pathological conditions, including autophagy regulation, protein degradation, and tumor progression [12–15]. OTUD7B (ovarian tumor domain-containing 7B), belonging to the OTU family, has been related to various pathologies. For example, OTUD7B activates mTORC2 by deubiquitinating and removing polyubiquitin chains from GβL, promoting its interaction with SIN1, which enhances AKT signaling and thereby contributes to lung tumorigenesis [12]. Moreover, OTUD7B negatively regulates antiviral immunity by deubiquitinating p62, promoting IRF3 autophagic degradation and balancing type I interferon signaling during viral infection [16]. Furthermore, OTUD7B regulates myocardial fibrosis following myocardial infarction by surpressing FAK and ERK/P38 signaling, suggesting it as a potential therapeutic target [17]. Screening a large collection of recombinant deubiquitinases with deubiquitin probes identified a subfamily with OTU domains that show ubiquitin linkage specificity [18]. As for OTUD7B, it preferentially disassembles both K11-, K48-, K63-linked ubiquitin chains [19]. Recently, OTUD7B was found to play a role in modulating cardiac remodeling via removing K48-linked ubiquitin chains, thereby stabilizing KLF4 [20]. However, the relationship between OTUD7B and both fatty acid metabolism and regulated cell death remains unclear. Given the critical role of these processes in cardiac hypertrophy [21, 22], it is essential to investigate the impact of OTUD7B on fatty acid metabolism and regulated cell death.

Mitochondrial fatty acid oxidation (FAO) is responsible for producing the majority of adenosine triphosphate (ATP), supplying over 70% of the energy required for cardiac metabolism under normal conditions [23]. However, during onset of cardiac dysfunction caused by pressure overload, the FAO rate commonly declines [24]. This reduction is linked to transcriptional alterations in genes that encode key enzymes involved in FAO [25]. To date, research have highlighted the importance of preserving FAO homeostasis to improve cardiac function under pressure overload [26, 27]. Previous studies have reported HNF4α as a key regulator of metabolic homeostasis. It drives the transcription of genes involved in intestinal and cardiac lipid metabolism, thereby regulating intestinal stem cell regeneration and cardiac remodeling [28, 29]. Additionally, impaired FAO leads to accumulation of partially oxidized fatty acids, promoting lipid peroxide production and triggering ferroptosis [30]. This novel form of regulated cell death, characterized by excessive lipid peroxide accumulation [22], has an increasingly recognized role in exacerbating cardiac hypertrophy and heart failure [31, 32]. These above studies suggest that modulating FAO may alleviate ferroptosis and improve cardiac dysfunction. However, the specific upstream regulatory mechanisms driving this process in pathological cardiac hypertrophy require further exploration.

This study demonstrates that OTUD7B protein expression is reduced in cardiomyocytes treated with phenylephrine (PE) and in myocardium subjected to transverse aortic constriction (TAC). OTUD7B deficiency worsens pathological cardiac hypertrophy and dysfunction, whereas its overexpression mitigates hypertrophy. Mechanistically, OTUD7B interacts with HNF4α and deubiquitinates it at the K48 site, thereby stabilizing its expression. This stabilization upregulates fatty acid metabolism and inhibits ferroptosis, ultimately alleviating cardiac hypertrophy. In summary, our findings identify OTUD7B as a potential candidate for treating cardiac hypertrophy and highlight the promise of modulating FAO via an HNF4α-targeted strategy.

## Methods

### Animals and experimental procedures

All animal experiments were performed in strict adherence to the guidelines for laboratory animal care and were approved by Nankai University Ethics Committee on Animal Care (approval no.2022-SYDWLL-000273).

All animals were housed in a specific pathogen-free (SPF) environment with stable conditions, maintaining a temperature range of 20–24 °C, humidity of 30–70%, and a 12-h light/dark cycle. The mice had unrestricted access to food and water and were acclimated for at least one week before study began.

To induce cardiac hypertrophy through pressure overload, male mice aged 8–10 weeks underwent TAC. Anesthesia was administered via intraperitoneal injection using 2% tribromoethanol (T48402, Sigma-Aldrich, USA). A thoracotomy was performed to expose the thoracic aorta, which was then constricted using a blunt 27-gauge needle and secured with a 6–0 nylon suture. The needle was removed afterward, and the success of the procedure was confirmed via Doppler echocardiography. Following the TAC procedure, the skin was sutured, and then mice were transferred to a 37 °C incubator until awakening. Mice in sham group received identical surgery, except without aortic ligation. For postoperative analgesia, buprenorphine (0.1 mg/kg) was administered postoperatively for 7 days. All mice were sacrificed 4 weeks after the TAC or sham procedure for further examination. The heart was explanted for further molecular and histological assays, and the tibia was removed to measure its length.

To specifically knockdown OTUD7B in cardiomyocytes, we infected male mice with adeno-associated virus serotype 9 (AAV9) which carried a cardiac-specific promoter cTNT (cTNTpMCS-3Flag-T2A-EGFP, GV571, Genechem, Shanghai, China) and encoded either an empty vector (AAV9-shNC) or OTUD7B (AAV9-shOTUD7B). Mice were then divided into the following groups: 1) Sham + AAV9-shNC; 2) TAC + AAV9-shNC; 3) Sham + AAV9-shOTUD7B; and 4) TAC + AAV9-shOTUD7B. The mice received a tail vein injection of AAV9 (5 × 10^11^vg/mouse) for 1 month, followed by sham or TAC procedure for 4 weeks.

### Echocardiography

To measure cardiac function, mice were anesthetized with 1.5% isoflurane and positioned for imaging. Body hair in the left precordial area was shaved, and echocardiography was performed using a high-resolution small animal ultrasound system (VEVO 2100, FUJIFILM Visual Sonics) with a 30-MHz (MS400) probe. Both long- and short-axis structures of the heart were visualized. M-mode echocardiography captured parameters including left ventricular end systolic dimension (LVESd), left ventricular end diastolic dimension (LVEDd), left ventricular posterior wall (LVPW), interventricular septal thickness at diastole (IVSD), ejection fraction (EF), and fractional shortening (FS) derived from five consecutive cardiac cycles.

### Histological analysis

For histological evaluation, myocardial tissues were paraffin-embedded and sliced into 5 μm sections. Hematoxylin and eosin (H&E) staining (G1120, Solarbio, Beijing, China) was applied to reveal cardiomyocyte cross-sectional area. In addition, Sections embedded in OCT were stained with WGA-fluorescein isothiocyanate (GTX01502, GeneTex, USA) for cardiomyocyte area analysis, following manufacturer instructions. Masson’s trichrome (G1340; Solarbio, Beijing, China) and Sirius Red stain (S8060; Solarbio, Beijing, China) were conducted to assess fibrosis. Data were analyzed using ImageJ (National Institutes of Health). For each staining, three to six sections from each group and six randomly chosen fields were examined by a blinded investigator.

### Immunohistochemistry

Serial sections were deparaffinized, blocked with phosphate-buffered saline (PBS) containing 5% (v/v) normal goat serum and 1% (w/v) BSA, and incubated overnight at 4 °C in a humidified environment with rabbit anti-ACOT1 (1:100; Abmart, ps18574), anti-4-HNE (1:100; Abcam, ab46545) and anti-ACOT1. Next day, sections were incubated for 1 h at room temperature with HRP-conjugated anti-rabbit secondary antibody (1:500, Proteintech, SA00001-2). Isotype IgG control slides were also prepared to confirm antibody specificity for each staining.

### Cell culture and transfection

Neonatal rat ventricular myocytes (NRVMs) were obtained from 1- to 3-day-old Sprague–Dawley rats as previously described [33]. Briefly, the hearts were harvested in sterile conditions, washed with PBS three times, and digested with trypsin and collagenase. Fibroblasts were separated using differential adhesion method, and the remaining nonadherent cells were maintained in Dulbecco’s modified Eagle’s medium (DMEM, Gibco, USA) containing 1% penicillin/streptomycin (100 U/mL penicillin and 100 mg/mL streptomycin), 10% fetal bovine serum (FBS, Procell, Wuhan, China), 10 mM HEPES and 0.1 mM 5-bromo-2'-deoxyuridine (BrdU, Sigma-Aldrich, USA). After 48 h of culture, cardiomyocytes exhibiting synchronized pulsation were used in following experiments.

To silence the expression of OTUD7B, NRVMs were treated with 50 nM rat OTUD7B siRNA or 50 nM scramble siRNA as a control, following the manufacturer's instructions (OBiO Technology, Shanghai, China). Each well received 5 μL of Lipo3000 (GK20006, GlpBio, USA). After 6 h, the medium was replaced with a maintenance medium containing 1% FBS. Total RNA was isolated 48 h later for PCR analysis, and proteins were collected 72 h post-transfection for western blot analysis.

To overexpress the expression of OTUD7B and HNF4α, plasmids encoding full-length OTUD7B, HNF4α were cloned from cDNAs and ligated to pcDNA3.1 vector (OBiO Technology, Shanghai, China). The recombinant adenoviral vector pcADV-EF1-mNeonGreen-CMV-MCS-3xFLAG (OBiO Technology, Shanghai, China), and pcADV-EF1-mNeonGreen-CMV-MCS-6xHis (OBiO Technology, Shanghai, China) were used to construct Ad-OTUD7B, Ad-HNF4α and corresponding negative control (Ad-GFP). At 50%−70% confluency, NRVMs were infected with either Ad-GFP or Ad-OTUD7B, Ad-HNF4α in a half-volume medium at a multiplicity of infection (MOI) of 50, respectively, at 37 °C for 4 h. After infection, the medium was replaced with fresh, full-volume culture medium. Eight hours later, cells were starved in 1% FBS for 12 h, then treated with 1 μM angiotensin II (Ang II) (HY-13948, MedChemExpress, USA) or 50 μM PE (GC17319, GlpBio, USA) or vehicle for another 24 h. Finally, proteins were extracted for analysis by western blot. Additionally, degradation levels of HNF4α were assessed followed by treatment with 100 μM cycloheximide (HY-12320, MedChem Express, USA) for 0, 3, 6, and 9 h in the NRVMs transduced with Ad-OTUD7B, Ad-HNF4α, or Ad-vector for 48 h.

### Transcriptome sequencing

For RNA sequencing, we began by extracting total RNA from samples, constructing a cDNA library, and sequencing on an Illumina Novaseq 6000 platform. Alignment of sequence fragments to the mouse reference genome (mm10/GRCm38) was completed with HISAT2 software (v2.2.1), and SAM tools were employed to convert the output to BAM format, preserving alignment details. Gene expression levels were quantified as fragments per kilobase of exon per million mapped fragments (FPKM) using StringTie with default parameters. Gene set enrichment analysis was performed for all genes. Genes with a fold-change > 2 or < 0.5 and an adjusted *P*-value < 0.05, identified using R package “DESeq2”, were considered significantly differentially expressed.

### Single-nucleus RNA sequencing analysis

The single-nucleus RNA (snRNA) dataset of the human heart was sourced from Figshare (https://doi.org/10.6084/m9.figshare.c.5777948.v2). This dataset contains cardiac interventricular septum (IVS) tissues from 10 hypertrophic cardiomyopathy (HCM) patients who underwent surgical myectomy and 3 healthy heart transplant donors. The analysis was performed using R (version 4.3.1) and the Seurat package (version 4.1.1). Cells with more than 500 genes, fewer than 5,000 genes, and less than 20% mitochondrial genes were retained for further analysis. Gene expression data were processed with the "NormalizeData" function, followed by scaling. The "vst" method was then applied to select the top 2,000 highly variable genes in each sample. Based on these genes, we conducted principal component analysis (PCA) and visualized significant principal components (PCs) using the ElbowPlot function. To account for batch effects across the two research groups, we utilized the Harmony package (version 0.1.0). Using 20 PCs, we performed UMAP analysis, classifying fifteen cell clusters with the "FindClusters" function at a resolution of 0.5. Differentially expressed genes in each cluster were identified using the "FindAllMarkers" function with a threshold of 0.25. Cell types were annotated based on the top five DEGs per cluster, referencing a previously published study and the CellMarker database (http://bio-bigdata.hrbmu.edu.cn/CellMarker/).

### Determination of cell surface area in vitro

The surface area of primary cardiomyocytes was measured using TRITC-labeled rhodamine phalloidin staining, following manufacturer’s guidelines (CA1610, Solarbio, Beijing, China). Nuclei were stained with DAPI solution (C0065, Solarbio, Beijing, China), and images were obtained using a fluorescence microscope. ImageJ was employed to measure the surface area of primary cardiomyocytes.

### Western blotting (WB)

Protein samples were obtained from myocardial tissues or cells by lysing with RIPA buffer (R0010, Solarbio, Beijing, China), supplemented with a protease inhibitor cocktail (HY-K0010, MedChem Express, USA). Equal amounts of protein were quantified using the BCA protein assay kit (PC0020, Solarbio, Beijing, China) and separated on a 10% SDS-PAGE gel, followed by transfer to PVDF membranes (IPVH00010, Millipore). The membranes were blocked with 5% skim milk at room temperature for 1 h to prevent nonspecific binding. Primary antibodies were then applied overnight at 4 °C, and after which the membranes were washed and incubated with HRP-conjugated secondary antibodies for 1.5 h at room temperature. Finally, chemiluminescence (ECL) reagent was applied, and images were acquired with ECL system. Primary antibodies: anti-OTUD7B (A17329, Abclonal, Wuhan, China), anti-HNF4α (A20865, Abclonal, Wuhan, China), anti-ACOT1 (ps18574, Abmart, Shanghai, China), anti-ACSL4 (R26735, Zenbio, Sichuan, China), anti-GAPDH (A19056, Abclonal, Wuhan, China), anti-Flag (AE063, Abclonal, Wuhan, China), anti-His (AE086, Abclonal, Wuhan, China), anti-HA (AE105, Abclonal, Wuhan, China).

### Detection of SOD, GSH, MDA and Fe^2+^ content

The mice plasma and NRVMs supernatant concentration of superoxide dismutase (SOD) (S0101M, Beyotime, Beijing, China), glutathione (GSH) (S0053, Beyotime, Beijing, China) and malondialdehyde (MDA) (S0131M, Beyotime, Beijing, China) were detected according to the manufacturers’ instructions. Fe^2+^ probes FerroOrange (E-BC-F101, Elabscience, Wuhan, China) were used to stain NRVMs to measure intracellular Fe^2+^ content. The stained cell fluorescence intensities were quantified on a Beckman flow cytometer and the data was analyzed with FlowJo software.

### Molecular docking (protein–protein docking)

The target proteins, OTUD7B and HNF4α, were identified in the Uniprot database (https://www.uniprot.org), corresponding to Uniprot IDs Q6GQQ9 and P41235, respectively. Their 3D structures were then docked through Protein–Protein docking module in Schrödinger.

### Co-immunoprecipitation (Co-IP)

Lysate samples were initially combined with specific antibodies and incubated on a rotating device at 4 °C for 4 h. This was followed by a second incubation period at the same temperature, where the samples were treated overnight with pre-cleared protein A/G agarose beads (sc-2001, SantaCruz). After centrifugation, three wash cycles were performed using PBS to remove non-specific proteins. Beads were then combined with 2 × SDS buffer and denatured at 95 °C for 10 min. Finally, the obtained protein sample was subsequently utilized for WB analysis.

### Quantitative real-time PCR

Total RNA was extracted from heart tissues and cells using TRIzol reagent (GK20008, GlpBio, USA) and subsequently reverse-transcribed into cDNA with the PrimeScript reagent kit (15662ES, Yeasen, China). For performing PCR, SYBR Green reagent kit (11201ES03, Yeasen, China) was used. Endogenous glyceraldehyde-3-phosphate dehydrogenase (GAPDH) served as internal reference gene. The 2^−ΔΔCT^ method was applied to determine the relative mRNA levels. Primers for target genes were synthesized according to the sequences listed in the supplementary Table S1 and obtained from Sangon Biotech.

### Transmission electron microscopy

To examine the ultrastructure of myocardium, ultrathin sections of cardiac tissue were prepared. Fresh heart tissue samples (1 mm^3^) were initially fixed with 2.5% glutaraldehyde at 4 °C overnight, followed by post-fixation with 1% osmium tetroxide. The specimens were then gradually dehydrated and embedded in LR White resin (Electron Microscopy Laboratory). Ultrathin sections (60 nm) were counterstained with uranyl acetate and then observed under a transmission electron microscope (Tecnai G2 Spirit BioTwin; FEI, Hillsboro, OR). Imaging and analysis were performed on at least five randomly selected fields.

### Statistical analysis

Statistical analyses in this study were conducted using GraphPad Prism 8.0 software. Results are presented as mean ± SEM. To detect significant differences, we applied a 2-tailed Student’s *t*-test for comparisons between two groups, and one-way ANOVA followed by Tukey post-hoc test for comparisons involving more than two groups. For repeated measurements from a single individual, each data set was analyzed using two-way repeated-measures ANOVA with pooled variance, and pairwise comparisons within each group were performed with a Tukey adjustment. A *P* value of less than 0.05 was deemed statistically significant.

## Results

### OTUD7B is downregulated in cardiac hypertrophy

**Fig. 1:**
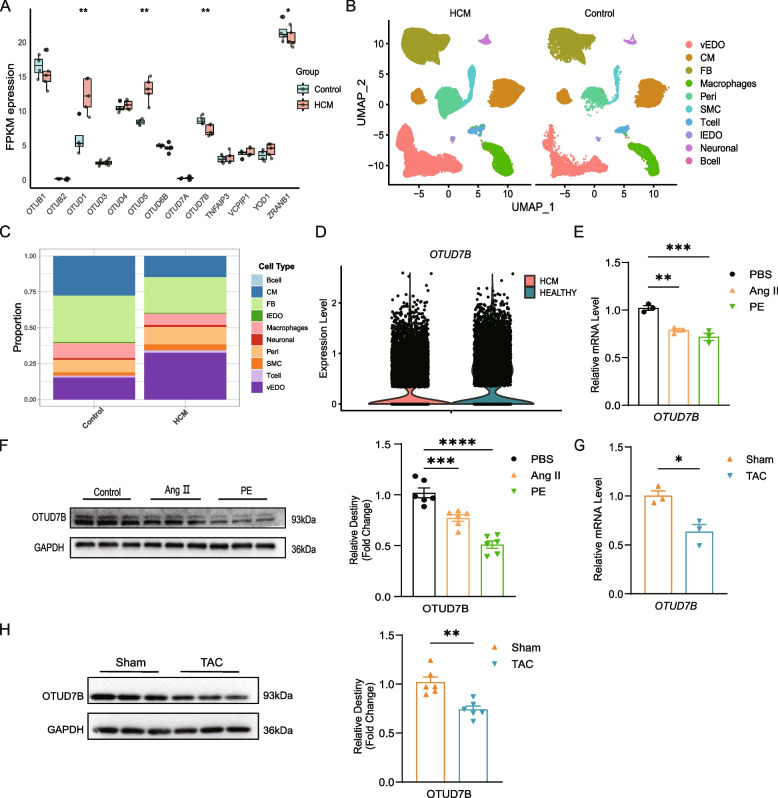
OTUD7B is downregulated in cardiac hypertrophy. **A** Boxplot showing the expression profile of DUBs from OTU family in GSE89714. **B** Single cell analysis showing the UMAP plot of cell distribution in HCM. **C** Single cell analysis showing the significant proportion change of cardiomyocytes between HCM and control group. **D** *OTUD7B* mRNA levels was downregulated in HCM samples. **E** qPCR analysis of the *OTUD7B* level in NRVMs treated with PBS, Ang II or PE for 24 h. **F** WB analysis of OTUD7B levels in NRVMs after 24 h of PBS, Ang II or PE treatment. **G** qRT-PCR analysis of the mRNA levels of *OTUD7B* in mice heart tissues at 4 weeks after TAC or sham surgery. **H** WB analysis of OTUD7B levels in mice hearts at 4 weeks after TAC or sham surgery (*n* = 6). Data are presented as mean ± SEM, Student’s *t*-test (**D**, **G**, **H**) and one-way analysis of variance (E) with Tukey post hoc test. Adjusted *p* values were provided in case of multiple groups. ns, *p* > 0.05; *p < 0.05; **p < 0.01; ***p < 0.001; ****p < 0.0001. OTUD7B, ovarian tumor domain-containing 7B; DUB, deubiquitinating enzyme; NRVMs, neonatal rat ventricular myocytes; HCM, hypertrophic myocardiopathy; Ang II, Angiotensin II; PE, phenylephrine; TAC, transverse aortic coarctation; WB, Western blotting

Initially, we investigated deubiquitinating enzymes within the OTU family across publicly available cardiac hypertrophy datasets. In the human cardiac hypertrophy dataset, GSE89714, OTUD7B and ZRANB1 was identified as the downregulated members of the OTU family (Fig. 1A). Similarly, analysis of the GSE221396 dataset, comprising three control and three Ang II-induced cardiac hypertrophy mice, revealed downregulation of OTUD7B (Figure S1A). Thus, OTUD7B was selected for further investigation. Subsequently, we examined OTUD7B’s cellular distribution at single-cell level using a human single-cell sequencing dataset containing samples from 10 cardiac IVS tissues of HCM patients and 3 IVS tissues from healthy heart transplant donors [34]. Our analysis confirmed that cardiomyocytes exhibited significant changes during the pathological progression of cardiac hypertrophy (Fig. 1B-C). Furthermore, OTUD7B was expressed in cardiomyocytes (Figure S1B), and its expression was lower in HCM patients compared to healthy controls (Fig. 1D).

NRVMs were treated with PE or Ang II to establish an in vitro model of myocardial hypertrophy. For OTUD7B, mRNA expression levels showed significant differences between PBS and PE or Ang II treatment (Fig. 1E), WB analysis further confirmed a notable reduction in OTUD7B protein levels in the myocardial hypertrophy models (Fig. 1F). Consistent findings were observed in wild-type mice subjected to TAC surgery, where OTUD7B expression was also significantly reduced (Fig. 1G-H).

### Cardiomyocyte-specific knockdown of OTUD7B exacerbates pressure overload-induced ventricular remodeling

**Fig. 2:**
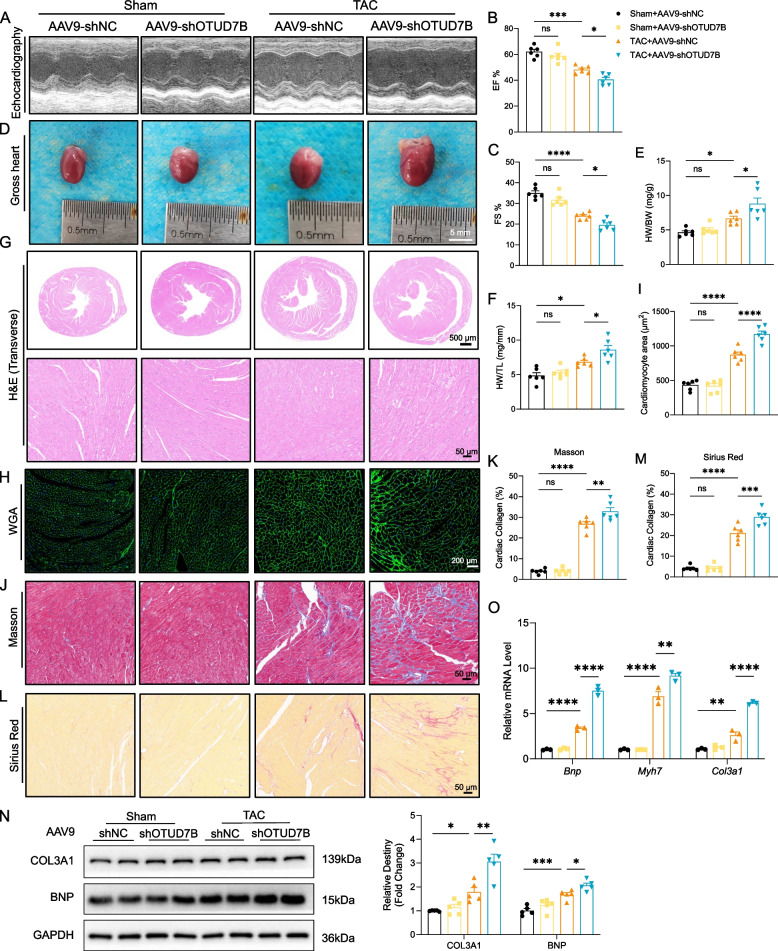
Cardiomyocyte-specific knockdown of OTUD7B exacerbates TAC-induced ventricular remodeling. **A** Representative images of echocardiography from each group. **B** and **C** Changes in ejection fraction (EF) (**B**) and fractional shortening (FS) (**C**) in each group. **D** Representative gross morphologies of the hearts (scale bar, 5 mm). **E** and **F** Changes in heart weight/body weight (HW/BW) (**E**) and heart weight/tibia length (HW/TL) (**F**) in each group. **G** to **I** Representative images of H&E staining (**G**), WGA staining (**H**), Masson staining (**J**, **K**), and Sirius Red staining (**L**, **M**) from each group (scale bar, 500 μm and 50 μm for H&E; 200 μm for WGA staining; 50 μm for Masson and Sirius Red staining). **O** The mRNA levels of *Bnp*, *Myh7* and *Col3a1* in myocardial tissues from each group. **N** Representative WB results of COL3A1 and BNP in myocardial tissues and density analysis. Data are presented as mean ± SEM, one-way analysis of variance (**B**, **C**, **E**, **F**, **I**, **K**, **M** to **O** with Tukey post hoc test. Adjusted *p* values were provided in case of multiple groups. ns, *p* > 0.05; *p < 0.05; **p < 0.01; ***p < 0.001; ****p < 0.0001. BNP, brain natriuretic peptide; H&E, hematoxylin–eosin

To elucidate the role of OTUD7B in ventricular remodeling, we administered AAV9-shOTUD7B to mice. Echocardiographic assessment revealed that OTUD7B knockdown exacerbated cardiac dysfunction following TAC, as indicated by reductions in EF and FS (Fig. 2A-C) and increases in LVESd and LVEDd (Figure S2C, D). Representative heart images further demonstrated enlarged hearts in OTUD7B knockdown mice compared to wild-type controls post-TAC (Fig. 2D). Body weight remained unchanged across the four groups (Figure S2A); however, heart weight, along with heart weight/body weight (HW/BW) and heart weight/tibia length (HW/TL) ratios, significantly increased in OTUD7B knockdown mice after TAC (Figure S2B, 2E, F). Histological examinations using H&E (Fig. 2G), WGA (Fig. 2H), Masson (Fig. 2J, K), and Sirius Red staining (Fig. 2L, M) consistently indicated that OTUD7B knockdown significantly intensified pressure overload-induced cardiac hypertrophy and fibrosis. Concordant with the histological and echocardiographic findings, protein levels of BNP (brain natriuretic peptide), COL3A1 (collagen type III alpha 1), and mRNA expressions of *Bnp*, *Myh7* (myosin heavy chain 7), and *Col3a1* were markedly elevated in OTUD7B knockdown mice post-TAC (Fig. 2N, O). Collectively, these findings demonstrate that OTUD7B knockdown exacerbates pressure overload-induced myocardial hypertrophy and fibrosis, leading to deteriorated cardiac function.

### OTUD7B alleviates PE-induced cardiomyocyte hypertrophy in vitro

**Fig. 3:**
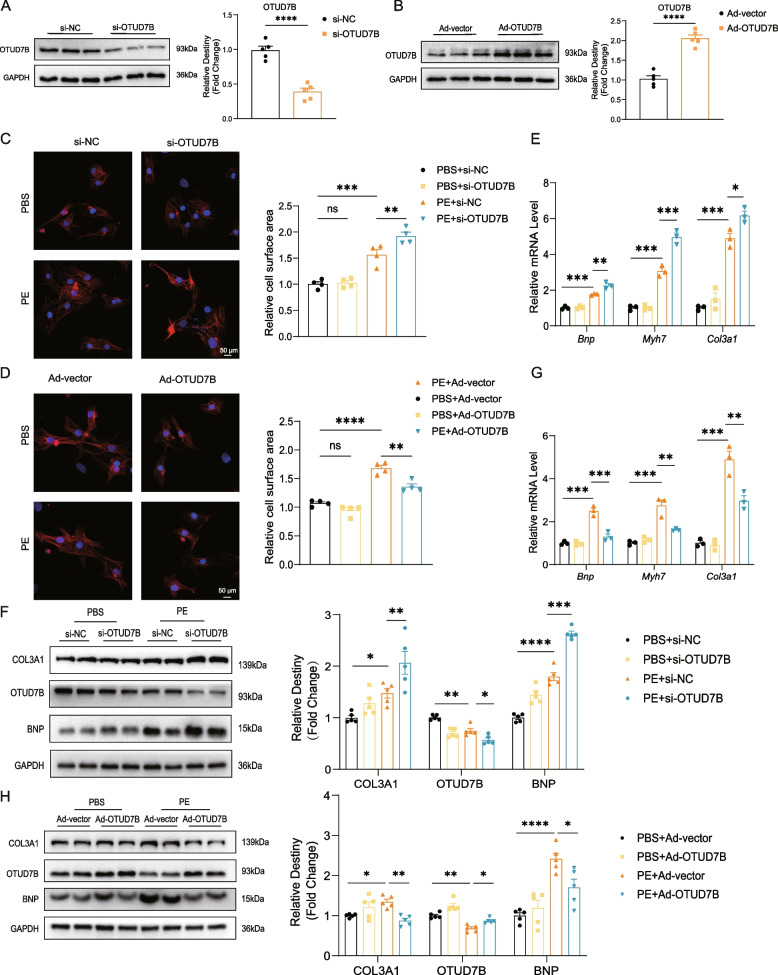
OTUD7B alleviates PE-induced cardiomyocyte hypertrophy in vitro. **A** and **B** Representative WB results of OTUD7B in NRVMs after transfected with si-OTUD7B (**A**) or Ad-OTUD7B (**B**). **C** and **D** TRITC Phalloidin staining and quantitative results of the cardiomyocyte size in NRVMs transfected with si-OTUD7B (**C**) or Ad-OTUD7B (**D**) and treated with PBS or PE for 24 h (scale bar, 50 μm). Cells were transfected with si-OTUD7B or Ad-OTUD7B or negative control before PE stimulation for 24 h. **E** and **G** The mRNA levels of *Bnp*, *Myh7* and *Col3a1* in NRVMs transfected with si-OTUD7B (**E**) and Ad-OTUD7B (**G**). **F** and **H** Representative WB results of OTUD7B, COL3A1 and BNP in NRVMs transfected with si-OTUD7B (**F**) and Ad-OTUD7B (**H**) and density analysis. Data are presented as mean ± SEM, student’s *t*-test (**A**, **B**) and one-way analysis of variance (**C** to **H**) with Tukey post hoc test. Adjusted *p* values were provided in case of multiple groups. ns, *p* > 0.05; *p < 0.05; **p < 0.01; ***p < 0.001; ****p < 0.0001

Given that OTUD7B knockdown exacerbates cardiac hypertrophy and its expression is reduced in myocardial tissue under TAC conditions, we subsequently explored the role of OTUD7B in modulating cardiac hypertrophy by influencing cardiomyocyte function iv vitro. To manipulate OTUD7B expression, we employed small interfering RNA (si-OTUD7B) to suppress OTUD7B expression and adenoviral vectors carrying OTUD7B-overexpressing plasmids (Ad-OTUD7B) to enhance its expression. The efficacy of OTUD7B inhibition and overexpression was confirmed by qRT-PCR and WB analysis (Figure S3A, B, Fig. 3A, B). Cardiomyocyte hypertrophy was induced using PE treatment. Consistent with our hypothesis, reduced OTUD7B expression under PE stimulation resulted in a significant increase in primary cardiomyocyte cell area, whereas OTUD7B overexpression effectively limited the enlargement of cardiomyocyte cell area (Fig. 3C, D). Furthermore, OTUD7B knockdown led to elevated mRNA levels of *Bnp*, *Myh7*, and *Col3a1*, along with increased BNP and COL3A1 protein expression, effects that were reversed upon OTUD7B overexpression. These findings indicate that OTUD7B effectively mitigates PE-induced pathological myocardial hypertrophy (Fig. 3E, F, G, H).

### OTUD7B overexpression attenuates ferroptosis

**Fig. 4:**
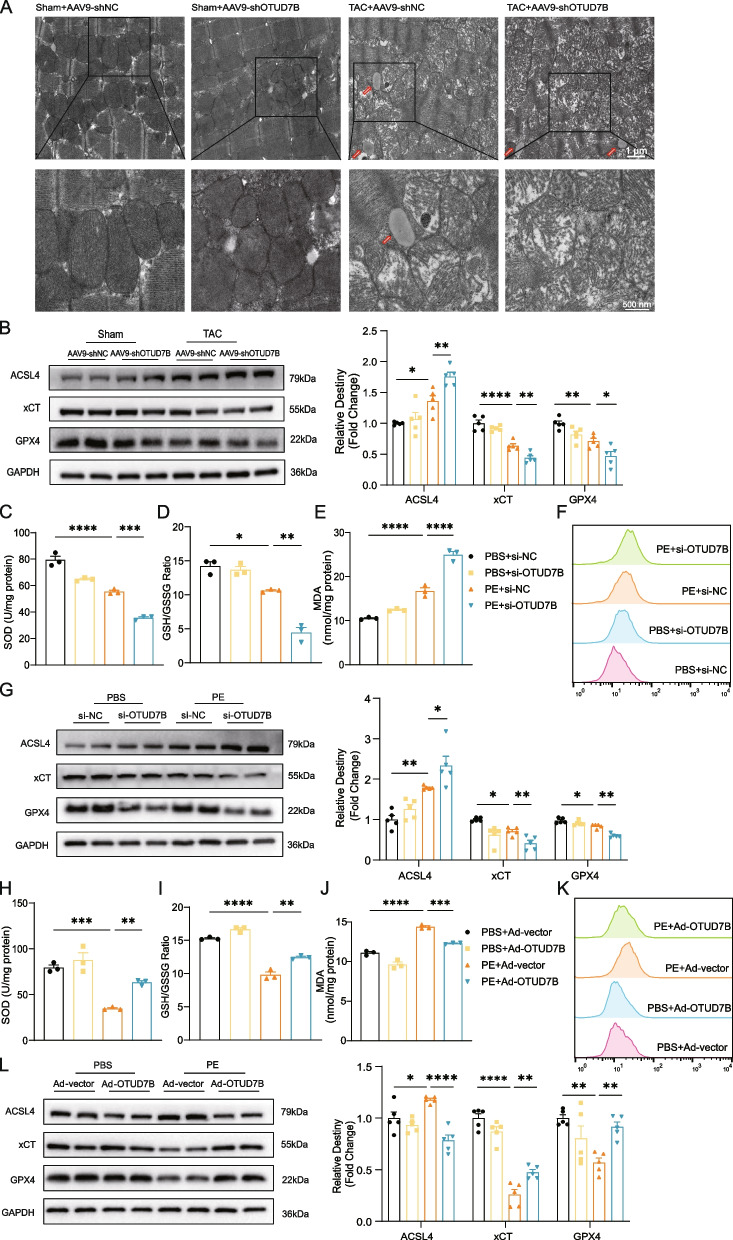
OTUD7B overexpression attenuates ferroptosis. **A** Representative transmission electron microscopy images of myocardial tissues from each group (scale bar, 1 μm, 500 nm). The red arrows indicate lipid droplets. **B** Representative WB results of ACSL4, xCT and GPX4 in myocardial tissues from each group and density analysis. (C to E) SOD (C), GSH/GSSG ratio (**D**), MDA (**E**) were measured in NRVMs transfected with si-OTUD7B before PE stimulation for 24 h. **F** Flow cytometry analysis of Fe^2+^ accumulation in NRVMs transfected with si-OTUD7B, followed by 24-h PE stimulation, and subsequently stained with FerroOrange (5 μM). **G** Representative WB results of ACSL4, xCT and GPX4 in NRVMs transfected with si-OTUD7B before PE stimulation for 24 h and density analysis. **H** to **J** SOD (**H**), GSH/GSSG ratio (**I**), MDA (**J**) were measured in NRVMs transfected with Ad-OTUD7B before PE stimulation for 24 h. **K** Flow cytometry analysis of Fe^2+^ accumulation in NRVMs transfected with Ad-OTUD7B, followed by 24-h PE stimulation, and subsequently stained with FerroOrange (5 μM). **L** Representative WB results of ACSL4, xCT and GPX4 in NRVMs transfected with Ad-OTUD7B before PE stimulation for 24 h and density analysis. Data are presented as mean ± SEM, one-way analysis of variance (**B** to **L**) with Tukey post hoc test. Adjusted *p* values were provided in case of multiple groups. ns, *p* > 0.05; *p < 0.05; **p < 0.01; ***p < 0.001; ****p < 0.0001

Myocardial ferroptosis is increased under TAC condition or PE stimulation, which manifests changed mitochondrial morphology and cristae structure and leads to cardiac dysfunction and ventricular remodeling [22, 31, 35]. Besides, excessive activation of oxidative stress induces lipid peroxidation, which plays a vital role in the initiation and progression of ferroptosis [36–38]. Transmission electron microscopy revealed that OTUD7B knockdown led to disrupted mitochondrial cristae and lipid droplet accumulation in myocardial tissue under TAC conditions (Fig. 4A). In hearts subjected to TAC, a significant reduction in SOD activity and GSH levels, an essential endogenous antioxidant, was observed. Knockdown of OTUD7B further exacerbated these effects (Figure S4A, B). Conversely, MDA, a biomarker of ferroptosis, was highest in OTUD7B-knockdown TAC hearts, indicating substantial lipid peroxidation (Figure S4C). 4-Hydroxy-2-nonenal (4-HNE), a reactive product of lipid peroxidation, acted as a biomarker for ferroptosis and showed a significant increase in OTUD7B-knockdown hearts following TAC surgery (Figure S4D). WB analysis of ferroptosis-related protein markers in cardiac tissue following TAC revealed an increase in pro-ferroptotic proteins, such as acyl-CoA synthetase long-chain family member 4 (ACSL4), which was further elevated by OTUD7B knockdown. Conversely, levels of anti-ferroptotic proteins, including solute carrier family 7 member 11 (SLC7A11, also known as xCT) and glutathione peroxidase 4 (GPX4), were reduced after TAC and further diminished with OTUD7B knockdown (Fig. 4B). In vitro, OTUD7B knockdown yielded similar results, marked by decreased SOD activity, reduced GSH levels, increased MDA content, elevated ACSL4 protein expression, and reduced xCT and GPX4 protein expression (Fig. 4C, D, E, G). Fe^2+^ ions overload disrupts the mitochondrial oxidative phosphorylation pathway, resulting in excessive reactive oxygen species (ROS). This oxidative stress promotes the peroxidation of unsaturated fatty acids in the cell membrane, compromising cellular structure and function and ultimately triggering ferroptosis [36, 39]. To further investigate this process, we assessed the intracellular Fe^2+^ ions levels using flow cytometry. The flow cytometry results showed that OTUD7B knockdown led to an increase in intracellular Fe^2+^ ions levels (Fig. 4F). Conversely, OTUD7B overexpression significantly reversed these adverse effects (Fig. 4H, I, J, K, L). These findings indicate that ferroptosis plays a critical role in cardiac hypertrophy, and OTUD7B exerts a protective effect against ferroptosis.

## OTUD7B knockdown causes a reprogramming of fatty acid metabolism in pathological cardiac hypertrophy

**Fig. 5:**
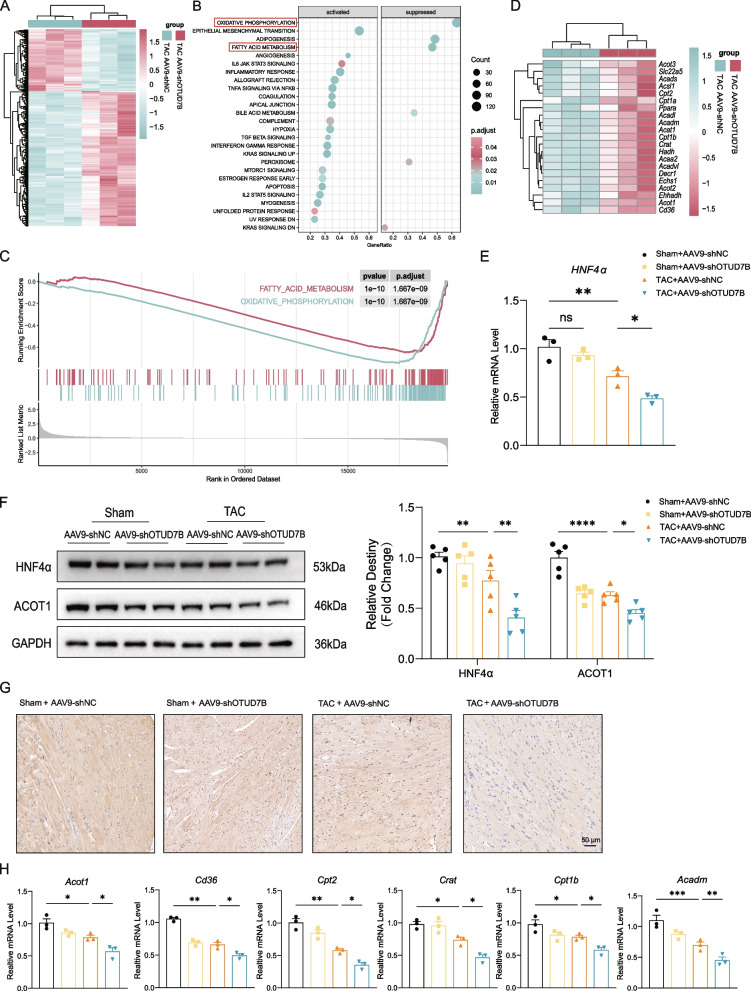
OTUD7B knockdown causes a reprogramming of fatty acid metabolic pathways in pathological cardiac hypertrophy. **A** Heatmap showing the altered gene expression profile of OTUD7B knockdown. **B** GSEA analysis of RNA-sequence data revealed several significantly changed processes in OTUD7B knockdown hearts. **C** GSEA analysis showing two significantly suppressed process, fatty acid metabolism and oxidative phosphorylation. **D** Heatmap showing the expression of genes involving in the fatty acid metabolism. **E** The mRNA levels of *HNF4α* in myocardial tissues from each group. **F** Representative WB results of HNF4α and ACOT1 in myocardial tissues from each group and density analysis. **G** Representative immunohistochemical staining showed the expression of ACOT1 in mice hearts tissues from each group (scale bar, 50 μm). **H** The mRNA levels of *Acot1*, *Cd36*, *Cpt2*, *Crat*, *Cpt1b* and *Acadm* in myocardial tissues from each group. Data are presented as mean ± SEM, one-way analysis of variance (**E**, **F** and **H**) with Tukey post hoc test. Adjusted *p* values were provided in case of multiple groups. ns, *p* > 0.05; *p < 0.05; **p < 0.01; ***p < 0.001; ****p < 0.0001. GSEA, gene set enrichment analysis

To explore the progressive alterations following OTUD7B knockdown, we then conducted transcriptome analysis to compare mRNA expression profiles in TAC-operated hearts of mice administered AAV9-shOTUD7B or AAV9-shNC. The Heatmap demonstrated that OTUD7B knockdown dramatically changed the gene expression profiles (Fig. 5A). GSEA analysis revealed that fatty acid metabolism and oxidative phosphorylation was the most significantly suppressed process in OTUD7B knockdown hearts (Fig. 5B, C). Furthermore, we examined the expression of genes involving in fatty acid metabolism process, including beta oxidation genes (*Acadvl*, *Acadm*, *Acads*, *Ehhadh*, *Hadh*, *Hadhb*, *Echs1*, *Decr1*), fatty acid activation genes (*Acot1*, *Acsl1*, *Acot2*, *Acot3*, *Acaa2*, *Acadl*), fatty acid transport genes (*Cpt1a*, *Cpt1b*, *Cpt2*, *CrAt*, *Slc22a5*) and fatty acid regulation genes (*Ppara*, *Cd36*). The heatmap revealed that these genes exhibited a reduction indicating a deteriorated fatty lipid metabolism (Fig. 5D). Acyl-CoA thioesterase 1 (ACOT1) is involved in the regulation of lipid metabolism by catalyzing the hydrolysis of long-chain acyl-CoAs into free fatty acids and coenzyme A. Downregulation of ACOT1 results in an increase in intracellular concentration of long-chain polyunsaturated fatty acids, thereby promoting ferroptosis [40]. Therefore, we hypothesize that a transcription factor may regulate the changes in ACOT1 and other lipid metabolism-related genes. Further investigation using the rVista2.0 database showed that majority of these genes contain binding sites for hepatocyte nuclear factor 4α (HNF4α) within their transcriptional regulatory regions (Table S2). Therefore, we examined HNF4α expression in TAC-induced cardiac hypertrophy. Both AAV9-shNC and AAV9-shOTUD7B mice showed significant reductions in HNF4α levels after TAC when compared to sham groups Moreover, OTUD7B knockdown mice exhibited a more robust decrease in HNF4α expression following TAC compared to AAV9-shNC mice (Fig. 5E, F). Regarding the expression of ACOT1 protein, similar results also was found (Fig. 5F, G). To evaluate whether the reprogramming of fatty acid metabolism in OTUD7B knockdown correlates with altered expression of HNF4α, qRT-PCR was performed to assess the expression of HNF4α target genes involved in FAO, including *Acot1*, *Cd36*, *Cpt2*, *Crat*, *Cpt1b*, and *Acadm* (coding for MCAD). These genes were downregulated by TAC surgery and further decreased their expression in the hearts of AAV9-shOTUD7B mice (Fig. 5H). These results indicate that OTUD7B knockdown leads to the downregulation of FAO-related genes, potentially due to the decreased level of HNF4α suppressing the transcription of FAO-related genes.

### OTUD7B mitigates dysregulation of fatty acid metabolism in PE-induced cardiac hypertrophy

**Fig. 6:**
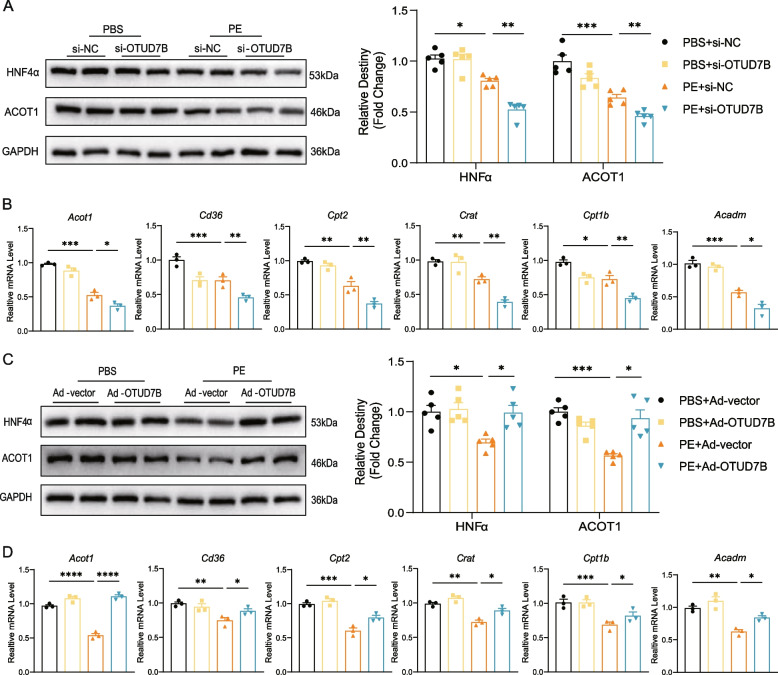
OTUD7B mitigates dysregulation of fatty acid metabolism in PE-induced cardiac hypertrophy. **A** Representative WB results of HNF4α and ACOT1 in NRVMs transfected with si-OTUD7B before PE stimulation for 24 h and density analysis. **B** The mRNA levels of *Acot1*, *Cd36*, *Cpt2*, *Crat*, *Cpt1b* and *Acadm* in NRVMs transfected with si-OTUD7B before PE stimulation for 24 h. **C** Representative WB results of HNF4α and ACOT1 in NRVMs transfected with Ad-OTUD7B before PE stimulation for 24 h and density analysis. **D** The mRNA levels of *Acot1*, *Cd36*, *Cpt2*, *Crat*, *Cpt1b* and *Acadm* in NRVMs transfected with Ad-OTUD7B before PE stimulation for 24 h. Data are presented as mean ± SEM, one-way analysis of variance (**A** to **D**) with Tukey post hoc test. Adjusted *p* values were provided in case of multiple groups. ns, *p* > 0.05; *p < 0.05; **p < 0.01; ***p < 0.001; ****p < 0.0001

Next, we knocked down and overexpressed OTUD7B in vitro respectively to investigate the impact of OTUD7B-induced changes in HNF4α expression and fatty acid metabolism. Following OTUD7B knockdown, the expression of HNF4α and ACOT1 was reduced (Fig. 6A). qRT-PCR showed that OTUD7B knockdown significantly downregulated the expression of FAO genes (*Acot1*, *Cd36*, *Cpt2*, *Crat*, *Cpt1b*, and *Acadm*) after PE stimulation (Fig. 6B). In contrast, overexpression of OTUD7B led to an increase in HNF4α and ACOT1 expression (Fig. 6C), and significantly upregulated the transcription of FAO genes (*Acot1*, *Cd36*, *Cpt2*, *Crat*, *Cpt1b*, and *Acadm*) after PE stimulation (Fig. 6D).

### OTUD7B directly interacts with HNF4α and regulates the stability of HNF4α protein through deubiquitination

**Fig. 7:**
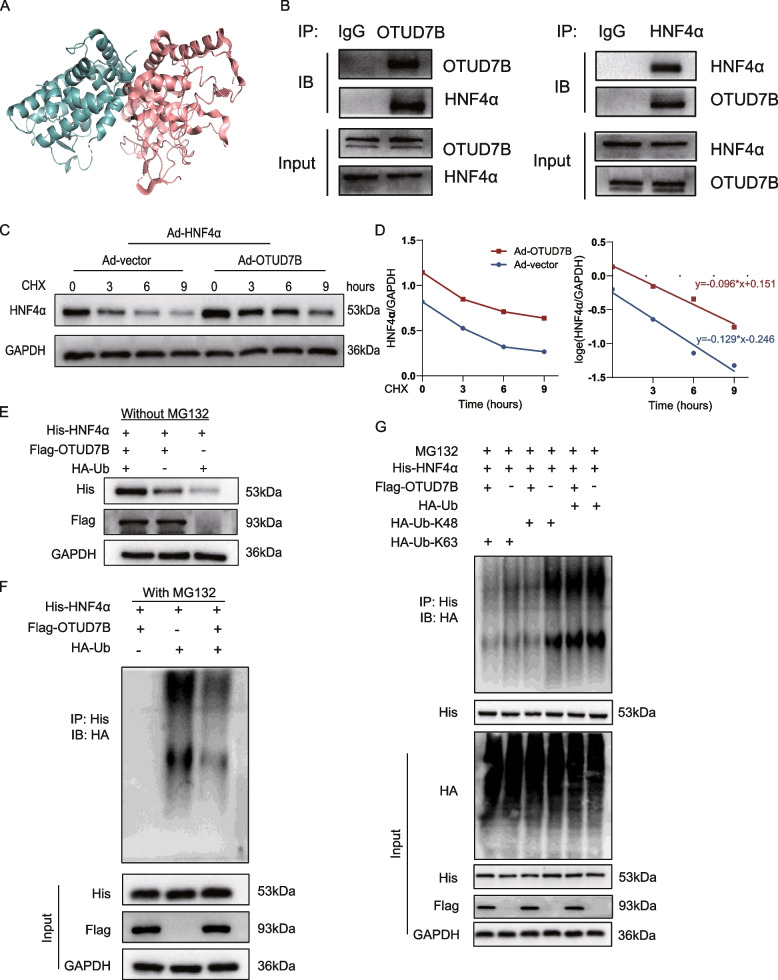
OTUD7B directly interacts with HNF4α and regulates the stability of HNF4α protein through deubiquitination. **A** Molecular docking results showing the possibility of OTUD7B interacts with HNF4α. **B** Representative endogenous Co-IP analyses showed the binding of OTUD7B to HNF4α in NRVMs under phenylephrine treatment for 24 h; IgG was used as the control. **C**, **D** NRVMs transduced with Ad-HNF4α, Ad-OTUD7B or Ad-vector for 48 h, followed by treatment with 100 μM cycloheximide for 3, 6 and 9 h. WB analysis of protein levels of HNF4α, GAPDH as the loading control. **E**, **F** Flag-OTUD7B, His-HNF4α and HA-Ub plasmids were transfected in HEK293T cell for 48 h. **E** Representative WB results of protein levels of Flag and His, GAPDH as the loading control. **F** 100 μM MG132 was added 6 h prior to protein lysate collection. Whole cell lysates were immunoprecipitated with His antibody, and then immunoblotted with antibodies against HA. **G** Immunoprecipitation of His-HNF4α in HEK293T cells that co-transfected with overexpression plasmids of Flag-OTUD7B, His-HNF4α, HA-Ub, HA-Ub-K48, and HA-Ub-K63 and then subjected to 100 μM MG132. Ubiquitinated HNF4α was detected by immunoblotting via using an His-specific antibody to clarify the ubiquitination pattern of HNF4α regulated by OTUD7B. CHX, cycloheximide;

To explore how OTUD7B regulates HNF4α, we conducted molecular docking, TransDSI, and Co-IP assays. We hypothesized that OTUD7B interacts with and deubiquitinates HNF4α. Molecular docking analysis indicated a potential interaction between OTUD7B and HNF4α, with a docking score of −258.68 and a confidence score of 0.8979 (Fig. 7A). TransDSI is an interpretable transfer learning framework based solely on protein sequences. It is pre-trained on a network of sequence similarities among 20,398 proteins and fine-tuned using 863 experimentally validated deubiquitinase-substrate interactions (DSIs). TransDSI was developed specifically to predict interactions between deubiquitinases and their substrates [41]. We used TransDSI to assess whether HNF4α is a deubiquitination substrate of OTUD7B. The TransDSI score for the OTUD7B-HNF4α interaction was 0.7979, suggesting a high probability that HNF4α is a substrate of OTUD7B. CoIP further confirmed these findings, demonstrating that OTUD7B directly interacts with HNF4α (Fig. 7B). NRVMs infected with Ad-OTUD7B and Ad-HNF4α were treated with cycloheximide (CHX) to inhibit protein synthesis. A time-dependent decrease in HNF4α protein level was observed, and OTUD7B overexpression slowed the degradation rate of HNF4α protein (Fig. 7C, D). Similarly, upon transfecting HEK293T cells with Flag-OTUD7B, His- HNF4α, and HA-Ub, we observed that OTUD7B stabilized HNF4α protein levels (Fig. 7E). To investigate whether OTUD7B reduces the ubiquitination of HNF4α, we treated cells with MG132, a proteasome system inhibitor. Our results demonstrated that OTUD7B markedly decreased the ubiquitination of HNF4α (Fig. 7F). Notably, in the presence of MG132, the protein level of HNF4α was not affected by OTUD7B, suggesting that OTUD7B inhibits HNF4α degradation via ubiquitin–proteasome system. As a deubiquitinating enzyme, OTUD7B removes ubiquitin chains from its target proteins. To further elucidate the regulatory mechanism of OTUD7B in HNF4α ubiquitination, we co-transfected OTUD7B, HNF4α, and mutant ubiquitin plasmids (retaining only K48 or K63 linkage sites) into HEK293T cells and treated them with MG132. Our findings revealed that OTUD7B significantly reduced HNF4α ubiquitination in cells expressing HA-Ub-K48 (Fig. 7G). These results indicate that OTUD7B stabilizes HNF4α by specifically cleaving its K48-linked ubiquitin chains.

### Ferrostatin-1 administration and HNF4α overexpression rescue the impact of OTUD7B knockdown on cardiac hypertrophy

**Fig. 8:**
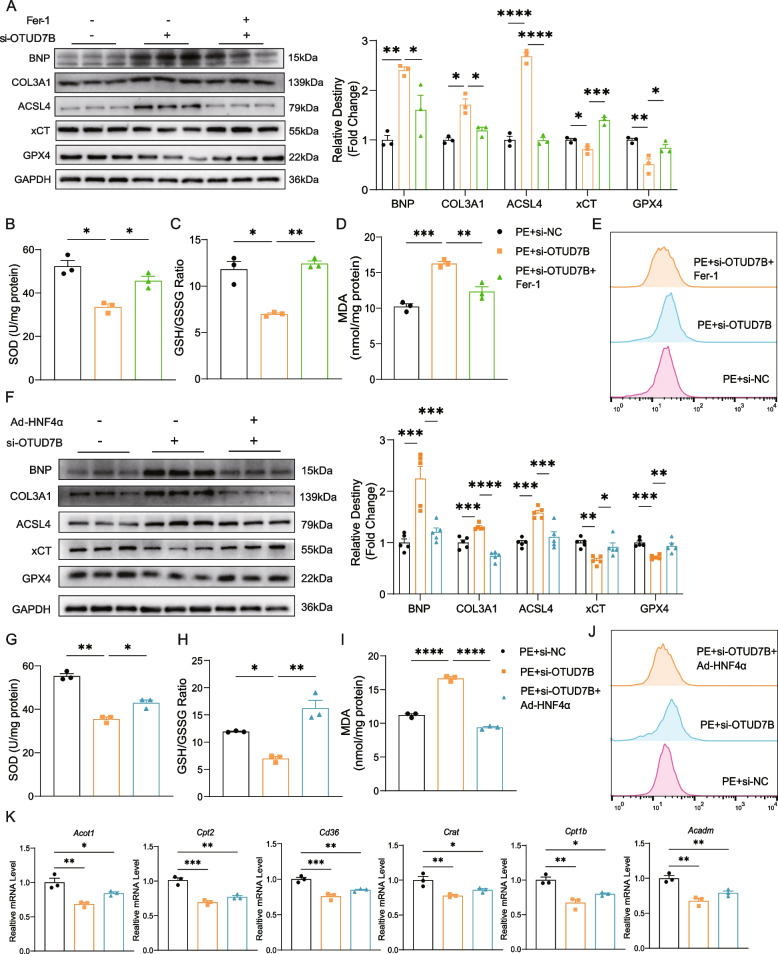
Ferrostatin-1 administration and HNF4α overexpression rescue the impact of OTUD7B knockdown on cardiac hypertrophy. **A** Representative WB results of BNP, COL3A1, ACSL4, xCT, and GPX4 in NRVMs transfected with si-OTUD7B or administered with Fer-1 (10 μM) before PE stimulation for 24 h and density analysis. **B** to **D** SOD (**B**), GSH/GSSG ratio (**C**), MDA (**D**) were measured in NRVMs transfected with si-OTUD7B or administered with Fer-1 before PE stimulation for 24 h. **E** Flow cytometry analysis of Fe^2+^ accumulation in NRVMs transfected with si-OTUD7B or administered with Fer-1 (10 μM), followed by 24-h PE stimulation, and subsequently stained with FerroOrange (5 μM). **F** Representative WB results of BNP, COL3A1, ACSL4, xCT, and GPX4 in NRVMs transfected with si-OTUD7B or Ad-HNF4α before PE stimulation for 24 h and density analysis. **G** to **I** SOD (**G**), GSH/GSSG ratio (**H**), MDA (**I**) were measured in NRVMs transfected with si-OTUD7B or Ad-HNF4α before PE stimulation for 24 h. **J** Flow cytometry analysis of Fe.^2+^ accumulation in NRVMs transfected with si-OTUD7B and Ad-HNF4α, followed by 24-h PE stimulation, and subsequently stained with FerroOrange (5 μM). **K** The mRNA levels of *Acot1*, *Cd36*, *Cpt2*, *Crat*, *Cpt1b* and *Acadm* in NRVMs transfected with si-OTUD7B and Ad-HNF4α before PE stimulation for 24 h. Data are presented as mean ± SEM, one-way analysis of variance (**A** to **K**) with Tukey post hoc test. Adjusted *p* values were provided in case of multiple groups. ns, *p* > 0.05; *p < 0.05; **p < 0.01; ***p < 0.001; ****p < 0.0001. Fer-1, ferrostatin-1

Next, we treated NRVMs with ferrostatin-1 (Fer-1), a well-established ferroptosis inhibitor, and found that its application significantly reduced protein levels of BNP and COL3A1. Additionally, there was an upregulation of the anti-ferroptotic proteins xCT and GPX4, alongside a reduction in the expression of the pro-ferroptotic protein ACSL4 (Fig. 8A). The levels of SOD and GSH also increased, while MDA levels decreased (Fig. 8B, C, D). The content of Fe^2+^ ions was reduced (Fig. 8E). Together, these findings indicate that Fer-1 treatment effectively mitigated ferroptosis, myocardial hypertrophy, and fibrosis resulting from OTUD7B knockdown. Similarly, simultaneous knockdown of OTUD7B and overexpression of HNF4α in NRVMs improved ventricular remodeling and fibrosis, evidenced by decreased BNP and COL3A1 expression (Fig. 8F). Myocardial ferroptosis was alleviated, as shown by downregulation of ASCL4, upregulation of xCT and GPX4 (Fig. 8F), increased SOD and GSH levels, reduced MDA levels and Fe^2+^ ions content (Fig. 8G, H, I, J). Additionally, HNF4α overexpression enhanced the expression of FAO genes (*Acot1*, *Cd36*, *Cpt2*, *Crat*, *Cpt1b*, and *Acadm*), rescuing the suppression of fatty acid metabolism induced by OTUD7B knockdown (Fig. 8K). These results indicate that the ferroptosis inhibitor Fer-1 and HNF4α overexpression can rescue adverse ventricular remodeling caused by OTUD7B knockdown.

## Discussion

Despite significant advancements in identifying molecular targets and signaling pathways involved in cardiac hypertrophy [42–45], current clinical pharmacological strategies for managing this condition remain inadequate. This study is the first to demonstrate that OTUD7B mitigates pathological cardiac hypertrophy by stabilizing HNF4α through deubiquitination. At the single-cell level, we observed downregulation of OTUD7B in hypertrophic cardiomyopathy patient samples. Bulk RNA-seq data from both Ang II-induced hypertrophic mice and hypertrophic cardiomyopathy patients further confirmed OTUD7B as one of the significantly downregulated members of the OTU family. Additionally, OTUD7B expression was markedly reduced in TAC-induced myocardial tissue and in NRVMs stimulated with Ang II. Our findings suggest that OTUD7B downregulation significantly suppresses fatty acid metabolism and promotes ferroptosis. Mechanistically, OTUD7B interacts with and stabilizes HNF4α through deubiquitination. These results highlight OTUD7B as a promising target for alleviating pathological cardiac hypertrophy.

DUBs modulate signaling pathways and prevent substrate protein degradation by altering ubiquitin linkage patterns. They play key roles in regulating cell signaling and protein interactions [46]. Understanding the regulatory mechanisms of DUBs in cardiac hypertrophy could reveal new therapeutic strategies. OTUD1 is upregulated in the heart under chronic Ang II stimulation, promoting pathological cardiac remodeling via STAT3 deubiquitination [43]. USP25 is elevated in hypertrophic myocardium, where it stabilizes SERCA2a by removing K48-linked ubiquitin chains, thereby inhibiting cardiac hypertrophy [44]. Additionally, USP28 and UCHL1 expression increases in hypertrophic and failing hearts [47, 48]. OTUD7B has been reported to regulate antiviral immunity [16], inflammation [49], and cancer cell proliferation [14]. It also contributes to post-infarction myocardial fibrosis by reducing α-SMA and collagen I expression via downregulation of FAK and ERK/P38 MAPK phosphorylation [17]. Du et al. reported that OTUD7B attenuates cardiac hypertrophy by inhibiting KLF4 ubiquitination and degradation [20]. Both their study and ours establish OTUD7B as a protective factor in cardiac hypertrophy. However, our study further links OTUD7B’s function to fatty acid metabolism and ferroptosis. Specifically, in our study, RNA sequencing revealed that OTUD7B downregulation is associated with alterations in fatty acid metabolism, oxidative phosphorylation, inflammatory response, angiogenesis, and the unfolded protein response. These findings provide a novel mechanistic perspective by incorporating metabolic dysfunction and ferroptosis into OTUD7B’s role in regulating cardiac hypertrophy regulation, beyond the previously identified KLF4-mediated pathway.

Increasing evidence indicates ferroptosis is pivotal in the pathophysiology of cardiac hypertrophy. One study demonstrated that FTH maintains cardiac homeostasis and alleviates left ventricular hypertrophy in mice through involvement of SLC7A11 and Fer-1, a ferroptosis inhibitor [31]. Another study reported that NDRG1 regulates ferroptosis and iron metabolism in cardiomyocytes, mitigating Ang II-induced ventricular hypertrophy and fibrosis [50]. GSH is a water-soluble tripeptide composed of glutamate, cysteine, and glycine. It exists intracellularly in either an oxidized (GSSG) or reduced (GSH) form and plays a critical role in maintaining cellular redox balance. its thiol group supports antioxidant properties and significantly influences lipid metabolism, which is particularly important in ferroptosis. GSH depletion reduces the activity of GPX4, impairing the GPX4-catalyzed reduction of lipid peroxides. Consequently, Fe^2^⁺ oxidizes lipids to generate ROS, thereby promoting ferroptosis [51]. In this study, OTUD7B knockdown resulted in decreased SOD levels, a reduced GSH/GSSG ratio, elevated Fe^2^⁺ content and concomitant downregulation of GPX4 protein, indicating that OTUD7B knockdown exacerbates ferroptosis.

Ferroptosis is fundamentally an oxidative damage process, primarily driven by mitochondrial changes due to the iron-dependent accumulation of lipid peroxidation products [52]. Transmission electron microscopy of myocardial tissue after OTUD7B knockout revealed disarranged mitochondria and lipid droplet accumulation in cardiomyocytes. OTUD7B knockdown also led to elevated levels of the oxidative products MDA and 4-HNE, both highly toxic to cells. MDA is commonly used as a biomarker to evaluate lipid peroxidation, whereas 4-HNE, a secondary metabolite produced during oxidation of polyunsaturated fatty acids (PUFAs), is considered the most cytotoxic aldehyde [53]. Excessive accumulation of MDA and 4-HNE are closely linked to cardiovascular diseases [54, 55]. Lipid peroxidation causes direct damage to cell membranes and is a defining factor in ferroptosis. The occurrence of ferroptosis depends on this peroxidation process, which requires the presence of PUFAs [56]. ACSL4 acylates free PUFAs to form PUFA-CoA, thereby promoting ferroptosis [57], while ACOT1 hydrolyzes acyl-CoA to release free fatty acids and CoA, regulating lipid metabolism. Previous studies have reported that ACOT1 is downregulated in the doxorubicin-induced cardiotoxicity (DIC) model, and overexpression of ACOT1 alters the composition of free fatty acids, inhibits lipid peroxidation, and exerts significant ferroptosis protection [58]. After OTUD7B knockdown, the expression of the ferroptosis marker ACSL4 was upregulated, while the expression of HNF4α and ACOT1 was downregulated. Additionally, the expression of fatty acid metabolism-related genes (*Cd36*, *Cpt2*, *Crat*, *Cpt1b*, and *Acadm*) was reduced, inhibiting fatty acid metabolism. Overexpression of HNF4α significantly alleviated the effects of OTUD7B knockdown, which exacerbated ferroptosis and suppressed fatty acid metabolism. These results suggest that OTUD7B regulates ferroptosis and fatty acid metabolism during cardiac hypertrophy through HNF4α.

During the progression of pathological cardiac hypertrophy, myocardial metabolism shifts from reduced FAO to increased reliance on glucose for energy, resulting in a decline in total ATP production. Maintaining normal mitochondrial FAO in cardiomyocytes may thus have protective effects. Consistent with this, our study demonstrates that HNF4α overexpression enhances the transcription of FAO-related genes during pathological cardiac hypertrophy, resulting in increased FAO and improved myocardial hypertrophy. HNF4α has been identified as a major regulator of metabolism, with functional binding sites in over 140 genes that are involved in glucose, lipid, and amino acid metabolism [59]. It controls the transcription of *Cpt1*, the rate-limiting enzyme in long-chain fatty acid oxidation, and also modulated genes related to acylcarnitine metabolism like *Cact*, *Cpt2*, and *Crat* [60–62]. In liver, HNF4α plays a critical role in maintaining metabolic homeostasis and lipid metabolism. Loss of HNF4α contributes to lipid accumulation, steatosis, and progression to fatty liver disease [63]. Post-translational modifications significantly affect HNF4α protein stability. High-fat diet feeding increases HNF4α acetylation, promoting its degradation [64], while SUMO2-mediated SUMOylation facilitates HNF4α degradation via the proteasome pathway [65]. In our study, we focused on the ubiquitination of HNF4α and revealed that OTUD7B stabilizes HNF4α by inhibiting its proteasomal degradation, thereby regulating its protein levels.

However, this study has several limitations. First, the downregulation of OTUD7B in hypertrophic cardiomyopathy samples was identified from publicly available single-cell sequencing data rather than clinically acquired data. Second, only male mice were used as experimental animals, without consideration of sex differences in cardiac hypertrophy [66]. Third, the study did not assess the effects of OTUD7B overexpression on ventricular remodeling in vivo. Lastly, the role of OTUD7B in fibroblasts and immune cells was not investigated.

In conclusion, this study provides robust evidence that OTUD7B-mediated deubiquitination of HNF4α is pivotal in reprogramming FAO and regulating ferroptosis during pressure overload-induced cardiac hypertrophy. These findings suggest that maintaining OTUD7B expression could be a promising therapeutic strategy for alleviating cardiac hypertrophy. Moreover, our investigation into lipid metabolism and peroxidation offers valuable insights into the complex interplay between lipid metabolism, lipid peroxidation, and ferroptosis.

## Supplementary Information

Additional file 1. Figure S1. (A) Boxplot showing the expression profile of DUBs from OTU family in GSE221396. (B) Single cell analysis showing the expression of OTUD7B across different cell clusters. Figure S2. (A) Changes in body weight of mice in each group. (B) Changes in heart weight of mice in each group. (C) Changes in left ventricular end diastolic dimension (LVEDd) of mice heart in each group. (D) Changes in left ventricular end systolic dimension (LVESd) of mice heart in each group. Figure S3. (A) The mRNA levels of OTUD7B in NRVMs transfected with si-OTUD7B. (B) The mRNA levels of OTUD7B in NRVMs transfected with Ad-OTUD7B. Figure S4. (A) SOD, (B) GSH/GSSG ratio, (C) MDA were measured in myocardial tissues from each group. (D) Representative immunohistochemical staining results indicating 4-HNE expression in mice hearts tissues from each group (scale bar, 50 μm).

Additional file 2.

## Data Availability

The data used in this study are available upon request from corresponding author.
