# Supplementary figures and images for: Deubiquitinase OTUD7B stabilizes HNF4α to alleviate pressure overload-induced cardiac hypertrophy by regulating fatty acid oxidation and inhibiting ferroptosis
Source: Biomark Res. 2025 Mar 29;13:53. doi: 10.1186/s40364-025-00766-2 (PMC11954242; doi:10.1186/s40364-025-00766-2)

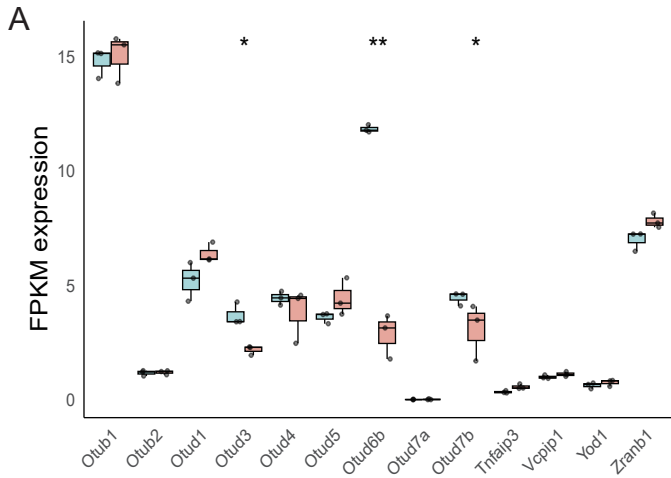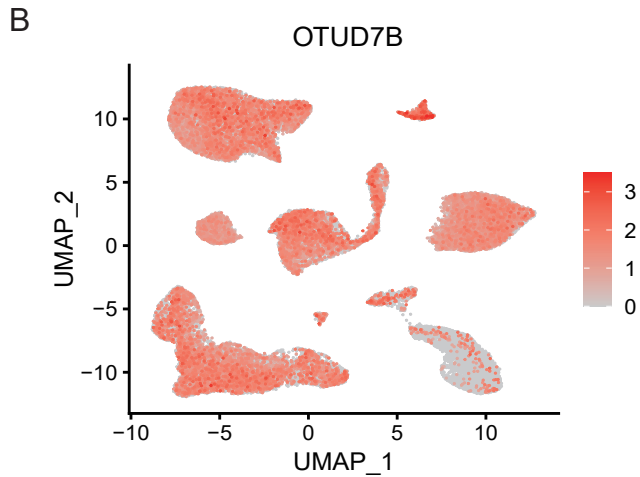

Supplement: Supplementary file 1 — Additional file 1. Figure S1. (A) Boxplot showing the expression profile of DUBs from OTU family in GSE221396. (B) Single cell analysis showing the expression of OTUD7B across different cell clusters. Figure S2. (A) Changes in body weight of mice in each group. (B) Changes in heart weight of mice in each group. (C) Changes in left ventricular end diastolic dimension (LVEDd) of mice heart in each group. (D) Changes in left ventricular end systolic dimension (LVESd) of mice heart in each group. Figure S3. (A) The mRNA levels of OTUD7B in NRVMs transfected with si-OTUD7B. (B) The mRNA levels of OTUD7B in NRVMs transfected with Ad-OTUD7B. Figure S4. (A) SOD, (B) GSH/GSSG ratio, (C) MDA were measured in myocardial tissues from each group. (D) Representative immunohistochemical staining results indicating 4-HNE expression in mice hearts tissues from each group (scale bar, 50 μm). [file 40364_2025_766_MOESM1_ESM.zip › Figure S1.pdf]

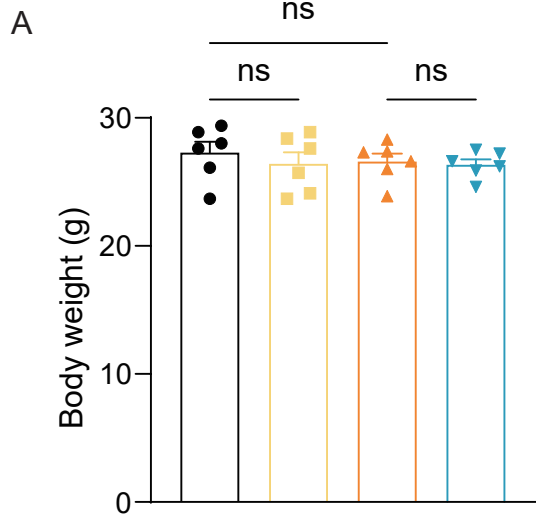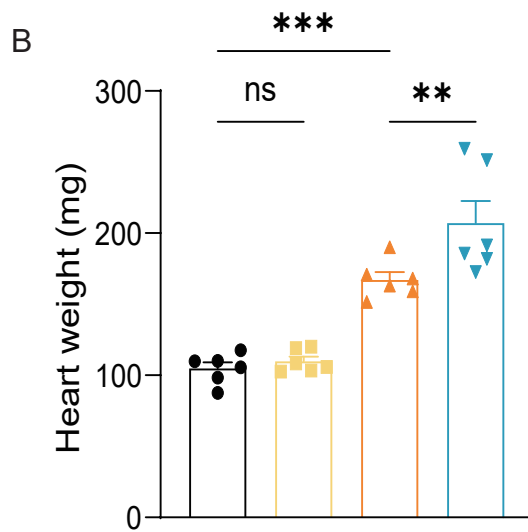

- Sham-AAV9-shNC
- Sham-AAV9-shOTUD7B
- ▲ TAC-AAV9-shNC
- ▼ TAC-AAV9-shOTUD7B

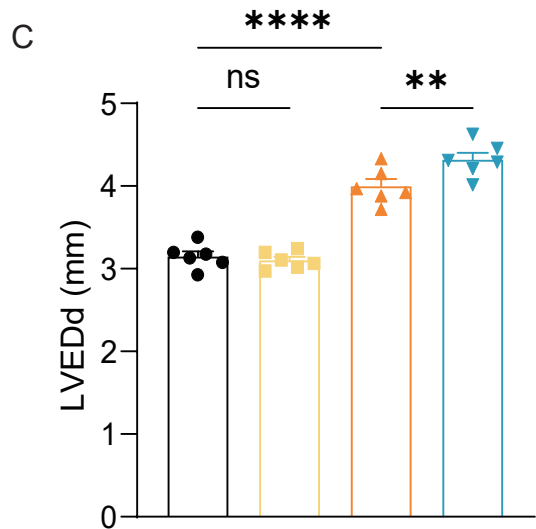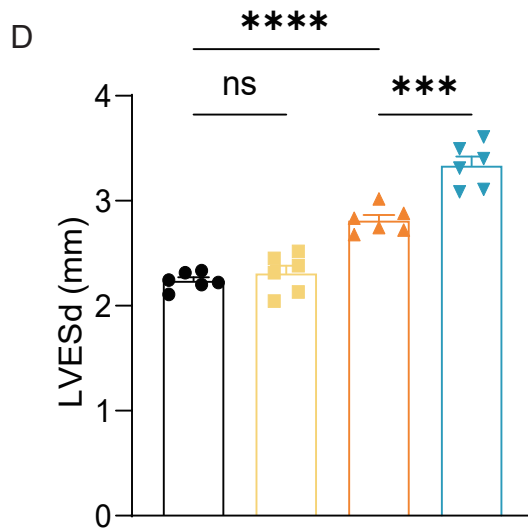

Supplement: Supplementary file 1 — Additional file 1. Figure S1. (A) Boxplot showing the expression profile of DUBs from OTU family in GSE221396. (B) Single cell analysis showing the expression of OTUD7B across different cell clusters. Figure S2. (A) Changes in body weight of mice in each group. (B) Changes in heart weight of mice in each group. (C) Changes in left ventricular end diastolic dimension (LVEDd) of mice heart in each group. (D) Changes in left ventricular end systolic dimension (LVESd) of mice heart in each group. Figure S3. (A) The mRNA levels of OTUD7B in NRVMs transfected with si-OTUD7B. (B) The mRNA levels of OTUD7B in NRVMs transfected with Ad-OTUD7B. Figure S4. (A) SOD, (B) GSH/GSSG ratio, (C) MDA were measured in myocardial tissues from each group. (D) Representative immunohistochemical staining results indicating 4-HNE expression in mice hearts tissues from each group (scale bar, 50 μm). [file 40364_2025_766_MOESM1_ESM.zip › Figure S2.pdf]

A

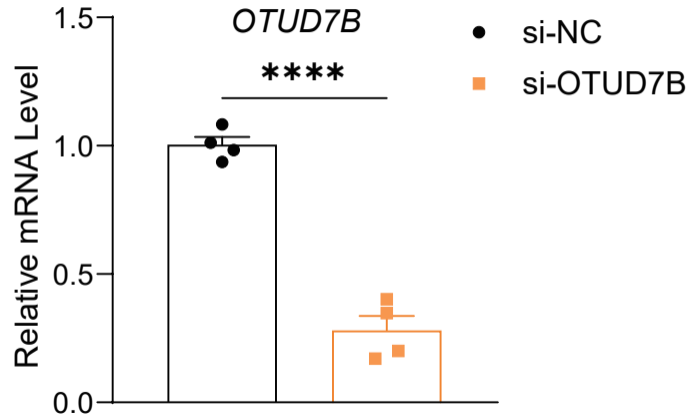

B

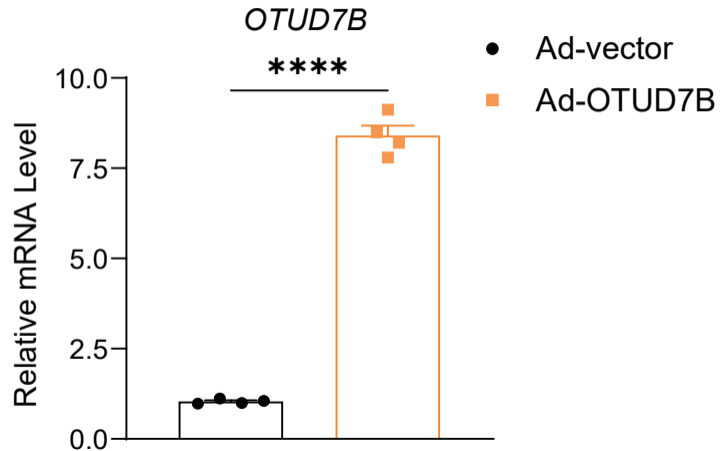

Supplement: Supplementary file 1 — Additional file 1. Figure S1. (A) Boxplot showing the expression profile of DUBs from OTU family in GSE221396. (B) Single cell analysis showing the expression of OTUD7B across different cell clusters. Figure S2. (A) Changes in body weight of mice in each group. (B) Changes in heart weight of mice in each group. (C) Changes in left ventricular end diastolic dimension (LVEDd) of mice heart in each group. (D) Changes in left ventricular end systolic dimension (LVESd) of mice heart in each group. Figure S3. (A) The mRNA levels of OTUD7B in NRVMs transfected with si-OTUD7B. (B) The mRNA levels of OTUD7B in NRVMs transfected with Ad-OTUD7B. Figure S4. (A) SOD, (B) GSH/GSSG ratio, (C) MDA were measured in myocardial tissues from each group. (D) Representative immunohistochemical staining results indicating 4-HNE expression in mice hearts tissues from each group (scale bar, 50 μm). [file 40364_2025_766_MOESM1_ESM.zip › Figure S3.pdf]

A

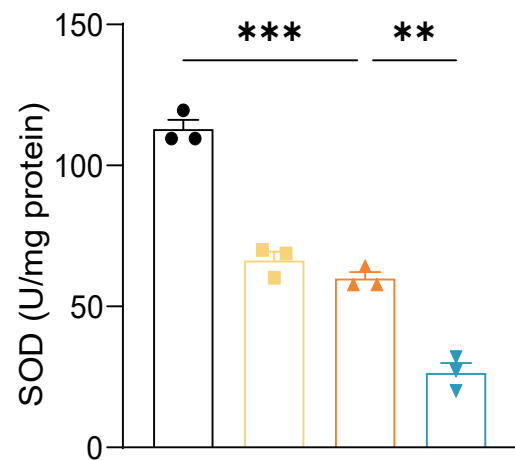

B

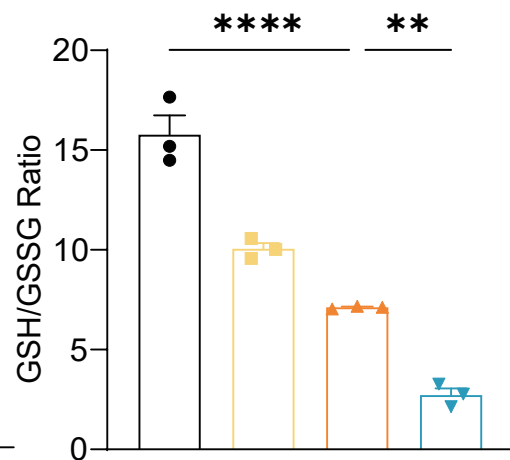

C

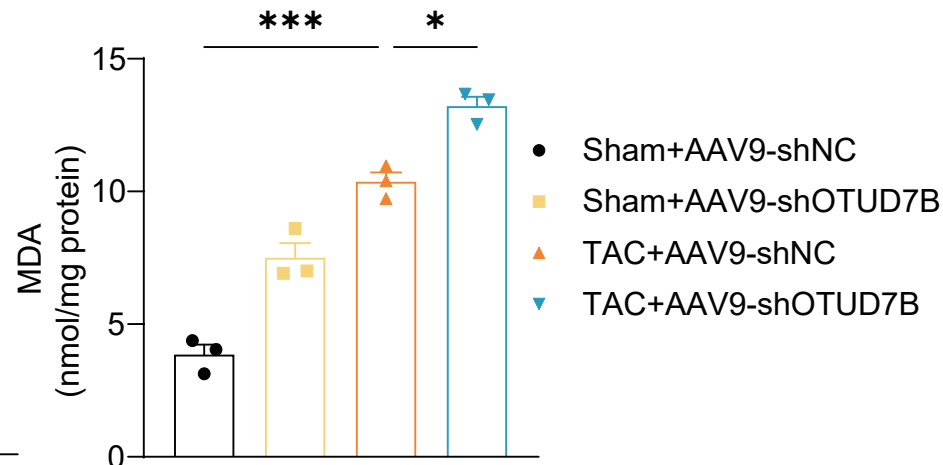

D

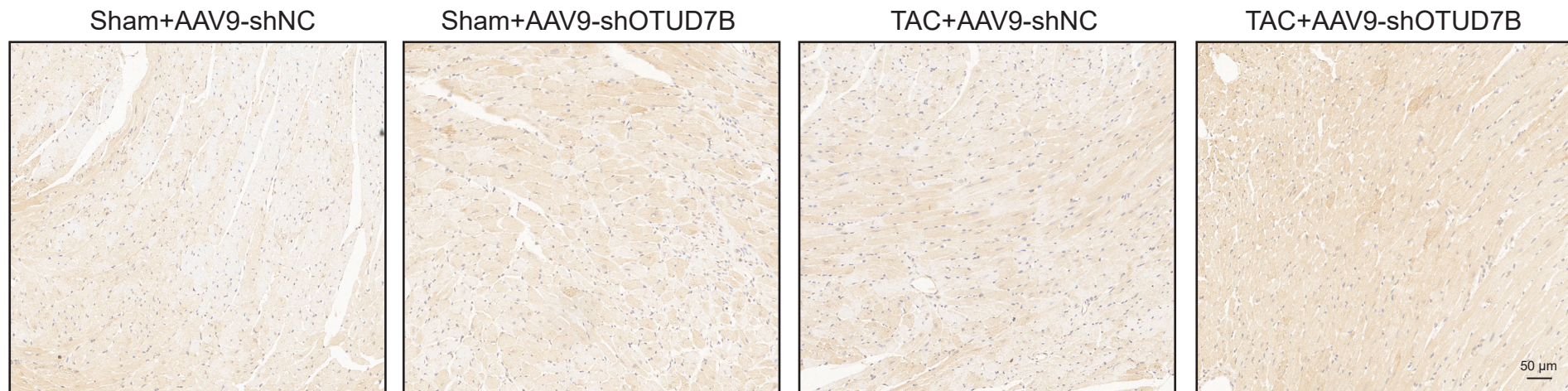

Supplement: Supplementary file 1 — Additional file 1. Figure S1. (A) Boxplot showing the expression profile of DUBs from OTU family in GSE221396. (B) Single cell analysis showing the expression of OTUD7B across different cell clusters. Figure S2. (A) Changes in body weight of mice in each group. (B) Changes in heart weight of mice in each group. (C) Changes in left ventricular end diastolic dimension (LVEDd) of mice heart in each group. (D) Changes in left ventricular end systolic dimension (LVESd) of mice heart in each group. Figure S3. (A) The mRNA levels of OTUD7B in NRVMs transfected with si-OTUD7B. (B) The mRNA levels of OTUD7B in NRVMs transfected with Ad-OTUD7B. Figure S4. (A) SOD, (B) GSH/GSSG ratio, (C) MDA were measured in myocardial tissues from each group. (D) Representative immunohistochemical staining results indicating 4-HNE expression in mice hearts tissues from each group (scale bar, 50 μm). [file 40364_2025_766_MOESM1_ESM.zip › Figure S4.pdf]
