# Supplementary material for: Deubiquitinase OTUD7B stabilizes HNF4α to alleviate pressure overload-induced cardiac hypertrophy by regulating fatty acid oxidation and inhibiting ferroptosis
Source: Biomark Res. 2025 Mar 29;13:53. doi: 10.1186/s40364-025-00766-2 (PMC11954242; doi:10.1186/s40364-025-00766-2)
Supplement: Supplementary file 2 — Additional file 2. [file 40364_2025_766_MOESM2_ESM.zip › Table S1.docx]

**Table S1.** Primers for qPCR detection.

| Gene name | Forward primer | Reverse primer |
| --- | --- | --- |
| Rat-*Otud7b* | TGCCTTCCAGCTTCCAGACCTC | ATTCAAACGCCCTGCCTGTTCC |
| Rat-*Bnp* | GCCAGTCTCCAGAACAATCC | GCTTGAACTATGTGCCATCTTG |
| Rat-*Myh7* | AACAGGCCAACACCAACCTG | CTACTCTTCATTCAGGCCCTTGG |
| Rat-*Col3a1* | ATAGCCACCCATTCCTCCG | CACCTGCTCCTGTCATTCC |
| Rat-*Cpt1b* | AGTGTGCCAGCCACAATTCA | ATAGGCTTCGTCATCCAGCAA |
| Rat-*Cpt2* | CTAAGAGATGCTCCGAGGCG | TCAAAGCCCTGGCCCATCG |
| Rat-*Cd36* | CCTCGGATGGCTAGCTGATT | TGTGGCCTGGTTCAACTAAT |
| Rat-*Crat* | CTTTCTACCAGCCAGGTCCC | TAGCAGATGCCGTAACCGTC |
| Rat-*Acot1* | GCCATCCAATCGGGATGTCT | AGTTCACTTCAGCGGGTCAC |
| Rat-*Acadm* | GGGGAAAGGCCAACTGGTAT | AGCCCCCATTGCAATCTTGA |
| Rat-*Gapdh* | GGCACAGTCAAGGCTGAGAATG | ATGGTGGTGAAGACGCCAGTA |
| Mus-*Otud7b* | AGCTCCAGCATTGTTTCCCT | CAATTCAGACGCCCTGCCTG |
| Mus-*Bnp* | CGGGCTGAGGTTGTTTTAGG | GCCGCAGGCAGAGTCAGA |
| Mus-*Myh7* | GCCCCAAATGCAGCCAT | CGCTCAGTCATGGCGGAT |
| Mus-*Col3a1* | ACGTAAGCACTGGTGGACAG | CCGGCTGGAAAGAAGTCTGA |
| Mus-*Hnf4α* | GTGCCAACCTCAATTCATC | GCTGCTCCTTCATAGACTC |
| Mus-*Cd36* | GGTCTATCTACGCTGTGTT | GTATGTCCTATGCTCATCTTC |
| Mus-*Cpt2* | ACCAGTGAGAACCGAGAT | CTAAGCAGAGGCAGAAGAC |
| Mus-*Acadm* | GGCGATGAAGGTTGAACT | CTCTGTGTTGAATCCATAGC |
| Mus-*Acot1* | GTAACTGTGCTGAGAATACTG | CTGGAACACGGTCTTGAG |
| Mus-*Crat* | CCTCTTACGCCATTGCTAT | CTGACACGGAGAAGTTGAT |
| Mus-*Cpt1b* | CACAGACAGAGGCACTTC | AGGAGACGGACACAGATAG |
| Mus-*Gapdh* | ACTCCACTCACGGCAAATTC | TCTCCATGGTGGTGAAGACA |
