# Supplementary material for: Deubiquitinase OTUD7B stabilizes HNF4α to alleviate pressure overload-induced cardiac hypertrophy by regulating fatty acid oxidation and inhibiting ferroptosis
Source: Biomark Res. 2025 Mar 29;13:53. doi: 10.1186/s40364-025-00766-2 (PMC11954242; doi:10.1186/s40364-025-00766-2)
Supplement: Supplementary file 2 — Additional file 2. [file 40364_2025_766_MOESM2_ESM.zip › Table S2.docx]

**Table S2.** *HNF4α* binding sites in transcriptional regulatory regions of genes in Fig5D.

| **Gene ID** | **Gene** | **Binding Sites for *HNF4α*** | |
| --- | --- | --- | --- |
|  |  | **Site 1** | **Site 2** |
| 12895 | *Cpt1b* | gTGACCTTTTccc |  |
| 12896 | *Cpt2* | aTGAACTTTTtca |  |
| 12491 | *Cd36* | gttCAAACTTCAc | attAAAAGTTCTt |
| 12908 | *Crat* | TGGCCCTCTGTCTc |  |
| 110446 | *Acat1* | tTTACCCTTGGCCt |  |
| 11364 | *Acadm* | ctgCAAAGACCAc |  |
| 171281 | *Acot3* | aggGAAAGGTCTAt | agtTAAAGATCAg |
| 26897 | *Acot1* | gTGACGCTTTGaaa |  |
| 15485 | *Hsd17b1* | gGACCTTTTGcet |  |
| 93898 | *Cers1* | gTGACCTTTTccc | cTGTATCTTTGgac |
